# Characterization of cardiac masses with T1 mapping

**DOI:** 10.1186/1532-429X-17-S1-Q32

**Published:** 2015-02-03

**Authors:** Shahryar G Saba, Patricia W Bandettini, Sujata M Shanbhag, Bruce S Spottiswoode, Peter Kellman, Andrew E Arai

**Affiliations:** 1 National Heart, Lung, and Blood Institute, Bethesda, MD, USA; 2 Cardiology, Hofstra North Shore-LIJ School of Medicine, Manhasset, NY, USA; 3 Cardiovascular MR R&D, Siemens Medical Solutions, Chicago, IL, USA

## Background

We hypothesize that T1 mapping contributes to the characterization of cardiac masses based on the spectrum of T1 relaxation times in tissue consisting of fat, calcium, melanin, blood and simple fluid. Given the well-established T1 lowering properties of melanin, T1mapping may assist in the evaluation of cardiac masses in patients with melanoma. Differentiating common cardiac tumors, such as myxomas, from thrombi with T1 mapping would also provide clinical utility, particularly in patients with a contraindication to gadolinium.

## Methods

We analyzed all CMR studies between January 2011 and April 2014 referred to our center for mass evaluation in patients with known metastatic melanoma, known or suspected thrombus, myxoma, lipoma, simple cyst and mitral annular calcification who underwent a modified look locker inversion recovery (MOLLI) T1 mapping sequence at 1.5T. We excluded 3 cases due to small mass size and poor differentiation of the mass from surrounding tissue. In the final analysis, we included 35 CMR examinations from 30 individual patients. More than one T1 measurement was performed in patients with multiple masses or for individual masses with T1 inhomogeneity. A total of 46 measurements were performed and averaged according to mass type. Melanomas were further subtyped as melanin-rich (T1 < 1000 ms) or melanin-poor (T1 >1000 ms).

## Results

Fat-based masses, melanin-rich melanomas and mitral annular calcification showed relatively short T1 times (Table and Figure). Conversely, thrombi and myxomas showed intermediate and relatively long T1 times, respectively, and the simple pericardial cyst showed the longest T1 time (Table and Figure).

**Figure 1:**
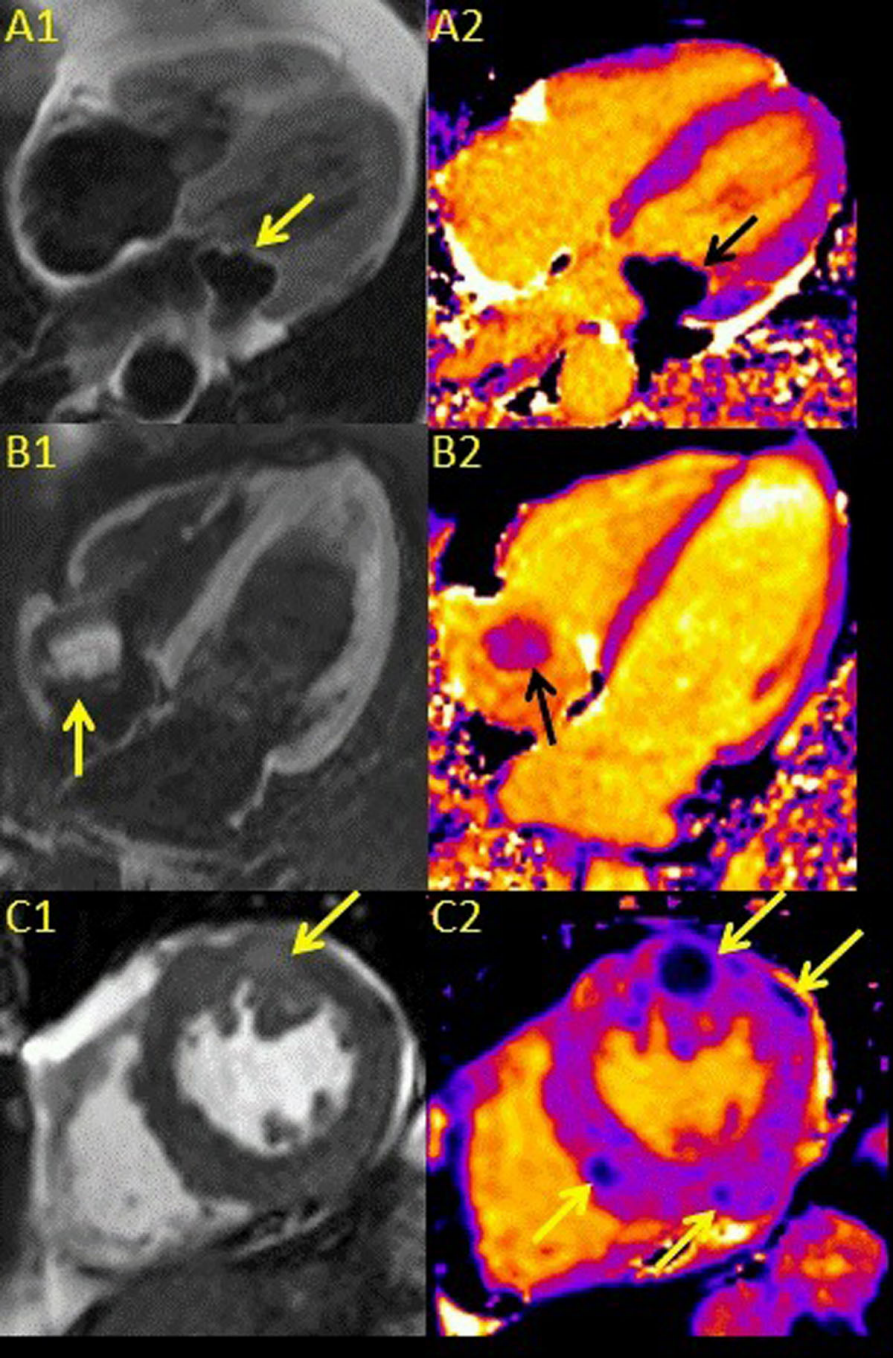
A1. A DIR TSE image demonstrates fat in the atrioventricular groove as well as a non-fat-based mass at the mitral annulus. A2. The corresponding T1 map shows a mass with a relatively low T1 value (417 ms) consistent with mitral annular calcification. B1. A DIR TSE SPAIR image demonstrates a catheter-related thrombus in the right atrium. B2. The T1 map shows a mass with an intermediate T1 time (1114 ms). C1. An SSFP image in a patient with metastatic melanoma demonstrates a region of bright signal intensity in the anterior myocardial segment. C2. The relatively low T1 values (~700 ms) of the melanin-rich metastatic foci on the corresponding T1 map facilitate identification of myocardium studded with tumor.

**Table 1:** T1 Values of Cardiac Masses/Tissues

Mass/Tissue	Mass/Tissue T1 Mean (ms)	Mass/Tissue T1 SD (ms)	CMR Examinations	Patients	Measurements
Fat-based	274.9	49.3	7	7	8

Calcium-based	416.9	NA	1	1	1

Melanoma (all)	893.0	237.9	11	6	17

Melanoma T1 < 1000 ms	765.2	145.4	8	3	12

Melanoma T1 > 1000 ms	1199.9	91.1	5	5	5

Thrombus	1044.7	256.9	11	11	15

Myxoma	1681.6	305.9	4	4	4

Simple pericardial cyst	3131.6	NA	1	1	1

Myocardium	1025.3	53.6	29	29	29

Epicardial fat	294.9	48.1	25	25	25

Blood	1575.2	291.5	27	27	27

## Conclusions

T1 mapping of masses may assist in tissue characterization, particularly in melanin-rich melanomas, myxomas and thrombi.

## Funding

This work was funded by the Division of Intramural Research, National Heart, Lung, and Blood Institute, National Institutes of Health, USA.

